# Factors influencing the accuracy of non-invasive blood pressure measurements in patients admitted for cardiogenic shock

**DOI:** 10.1186/s12872-019-1129-9

**Published:** 2019-06-18

**Authors:** Jitka Seidlerová, Pavlína Tůmová, Richard Rokyta, Milan Hromadka

**Affiliations:** 1 Internal Department II, University Hospital and Faculty of Medicine in Pilsen, Charles University, Pilsen, Czech Republic; 2 Biomedical Centre, Faculty of Medicine in Pilsen, Charles University, Pilsen, Czech Republic; 3 Cardiology Department, University Hospital and Faculty of Medicine in Pilsen and Faculty Hospital, Charles University, Pilsen, Czech Republic

**Keywords:** Blood pressure measurement, Auscultatory BP measurement, Oscillometric BP measurement, invasive BP measurement, cardiogenic shock

## Abstract

**Background:**

Although invasively measured blood pressure (invBP) is regarded as a “gold standard” in critically ill cardiac patients, the non-invasive BP is still widely used, at least at the initiation of medical care. The erroneous interpretation of BP can lead to clinical errors. We therefore investigated the agreement of both methods with respect to some common clinical situation.

**Methods:**

We included 85 patients hospitalized for cardiogenic shock. We measured BP every 6 h for the first 72 h of hospitalization, in all patients. Each set of BP measurements included two invasive (invBP), two auscultatory (auscBP), and two oscillometric (oscBP) BP measurements. InvBP was considered as a gold standard. Mean non-invasive arterial pressure (MAP) was calculated as (*diastolic pressure + (pulse pressure ÷ 3))*. We used Bland-Altman analysis and we calculated concordance correlation coefficients to assess agreement between different BP methods.

**Results:**

We obtained 967 sets of BP measurements. AuscMAP and oscMAP were on average only 0.4 ± 8.2 and 1.8 ± 8.5 mmHg higher than invMAP, respectively. On the other hand, auscSBP and oscSBP were on average − 6.1 ± 11.4 and − 4.1 ± 9.8 mmHg lower than invSBP, respectively. However, the mean differences and variability for systolic and diastolic BP variability were large; the 2 standard deviation differences were ± 24 and 18 mmHg. In hypotension, non-invasive BP tended to be higher than invBP while the opposite was true for high BP values. Clinical conditions associated with hypotension generally worsened the accuracy of non-invasive MAP.

**Conclusions:**

Mean arterial pressure measured non-invasively appears to be in good agreement with invasive MAP in patients admitted for cardiogenic shock. Several clinical associated with hypotension can affect accuracy of non-invasive measurement. Auscultatory and oscillometric measurements had similar accuracy even in patients with arrhythmia.

**Electronic supplementary material:**

The online version of this article (10.1186/s12872-019-1129-9) contains supplementary material, which is available to authorized users.

## Background

Invasively measured arterial blood pressure (invBP) measurement is an integral part of the management of critically ill patients and is considered to be the gold standard for blood pressure (BP) measurement. However, at least at the initiation of medical care (before other more urgent measures are taken), BP is measured non-invasively. Indeed, some data suggest that in real clinical practice it is not unusual for BP to be assessed non-invasively in critically ill patients [1]. We might presume that in low-income countries the use of invasive BP measurement in these patients is insufficient.

Several papers have reported data showing agreement between invBP and non-invasive auscultatory (auscBP) or oscillometric (oscBP) BP measurements in patients hospitalized in intensive care units. These reports included patients admitted for a variety of conditions (i.e., acute stroke, septic shock, major polytrauma, respiratory failure, etc.) as summarized by Lakhal [2]. However, very limited data are available regarding agreement between non-invasive and invasive BP measurement in acute non-surgical cardiac patients. Both invasive and non-invasive BP measurement methods have their limitations and possible sources of errors [2–4]. It is widely accepted that the oscillometric method is imprecise in cases of arrhythmia, although, some have reported the opposite [5]. Auscultatory measurement remains to be a ‘gold standard’ for non-invasive BP assessment however in real clinical practice (in intensive care units) it was practically replaced by oscillometric method.

Differences in BP measurement methods have the potential to impact clinical outcomes. In a large intensive care unit (ICU) database, severe, non-invasively assessed, systolic hypotension was associated with a significantly higher prevalence of acute kidney injury and ICU mortality than invasive systolic BP readings within the same BP range [6]. During non-cardiac surgery, Wax et al. [7] found differences in therapeutic interventions (e.g., blood transfusions and vasopressor infusions) relative to BP monitoring methods.

In the present study we therefore investigated how non-invasive BP measurements agree with invasive BP measurements and which clinical conditions or factors can influence the accuracy of auscultatory and oscillometric measurements in patients with cardiogenic shock.

## Methods

### Study population

This prospective study included 85 patients with cardiogenic shock hospitalized at the Coronary-Care Unit (CCU) of the University Hospital in Pilsen, Czech Republic between March 2017 and April 2018. We used following criteria for cardiogenic shock: (i) systolic blood pressure < 90 mmHg for > 30 min or vasopressors required to achieve a blood pressure ≥ 90 mmHg; (ii) pulmonary congestion or elevated left-ventricular filling pressures; (iii) signs of impaired organ perfusion with at least one of the following criteria: (a) altered mental status; (b) cold, clammy skin; (c) oliguria; (d) increased serum-lactate. We included patients in whom invasive blood pressure monitoring was indicated. None of the patients received an intra-arterial catheter only for the purpose of the study. The management of the patient was guided exclusively by invBP with the target invasive mean arterial pressure (invMAP) value ≥70 mmHg. Exclusion criteria were as follows: extreme obesity (body mass index > 40 kg/m^2^), use of extracorporeal membrane oxygenation, intra-aortic balloon counter-pulsation, and cannula insertion into other than the left or right radial artery.

### Blood pressure measurement

We started BP recordings as soon as the patient’s condition was stabilized (in all patients within three hours of the initiation of medical care).

### Order of BP measurements

First was the invasive BP value; next were two auscultatory BP measurements; followed by two oscillometric measurements; and lastly the second invasive BP measurement was recorded. For BP comparisons, the mean values of each pair of BP measurements were used. BP measurements were repeated in the same patient every six hours for 72 h; thus we expected to obtain12 sets of BP measurements for each patient (1020 sets of measurement). However, seven patients were dismissed from the CCU before end of the study. Moreover, in four cases we were not able to measure oscillometric BP. The values of invasive and auscultatory BP in these cases were: 108/79 and 110/70; 50/20 and 40/20; 105/78 and 110/70; 102/65 and 100/60 mmHg. In two times atrial fibrillation was present, in other two cases frequent supraventricular of ventricular extrasystoles were present. Therefore, number of BP sets totaled 967. In non-invasive measurements, systolic (SBP) and diastolic BP (DBP) were recorded and mean arterial pressure (MAP) calculated according to the formula: MAP = DBP + ((SBP-DBP) ÷ 3). For invasive measurements, we directly recorded MAP, SBP, and DBP. Only nine experienced nurses (i.e., shift supervisors) were responsible for taking all BP measurements. At the time of each BP measurement, the following were are also recorded: the patient’s Glasgow coma score, body temperature, electrocardiogram, Richmond Agitation-Sedation Scale (RASS), information on patient sedation, and whether the patient had received (was receiving) vasopressive or inotropic medications.

### Invasive blood pressure measurement

A 20-gauge cannula was inserted into the right or left radial artery and connected to a disposable pressure transducer (Combitrans Monitoring Set (arterial); B. Braun Melsungen AG, Germany) using 100-cm-long tubing. The transducer system was set up by an experienced nurse and checked by a physician in all cases. The forearm was at the same level as the brachial cuff (i.e., at the level of the phlebostatic axis) to eliminate hydrostatic pressure. Air bubbles were carefully flushed from the system before data collection. The zero level for the arterial blood pressure was taken at the right atrium (i.e., at the level of the phlebostatic axis) and the arterial waveform was displayed on a Solar 8000i monitor (GE Medical Systems Information Technologies Inc., WI, United States). The average resonance frequency of the catheter tubing-transducer system was 20 Hz (range, 16–25 Hz), combined with a damping coefficient of 0.3. In addition, the correct shape of the BP waveform was ascertained using the “fast flush” test. BP values were measured at the end of expiration assessed using capnography waveform and with the help of monitor freezing at the time of BP reading to minimize the effect of changing intrathoracic pressure on invasively measured BP values [3]. In patients with regular heart rhythm we recorded one invasive BP value just before and just after non-invasive BP was taken. In those cases when arrhythmia was present, we recorded three consecutive invasive BP values at the beginning and three consecutive invasive BP values at the end of BP measurement; from these values we calculated mean used in analysis.

### Non-invasive blood pressure measurement

A dual tonometer Nissei DM-3000 (NISSEI, Nihon Seimitsu Sokki Co., Japan) was used for taking non-invasive blood pressure measurements [8]. According to the manufacture’s recommendations, the patient’s mid-arm circumference was used to determine the appropriate brachial cuff size. The brachial cuff was placed on the same arm as the radial artery catheter. Two BP auscultatory measurements, followed by two oscillometric measurements were taken, with 1-min intervals between each measurement.

### Other measurements

Baseline biochemical variables (blood samples were drawn at the time of the first BP measurement) were measured. NT-pro BNP values were determined using original analytical kits from Roche on a Cobas 8000 analyzer. High-sensitivity cardiac troponin I (hsTnI) was measured using the Architect i2000 platform and a STAT High Sensitive Troponin-I assay (Abbott Diagnostics, USA). We measured serum lactate and blood pH initially, and 12, 24, and 48 h after admission to the CCU.

Left ventricle ejection fractions were recorded using bed-side echocardiograms, which were acquired using a Vivid 7 ultrasound system (GE Medical Systems, Horton, Norway) with a 3.4-MHz multi-frequency transducer. Left ventricular filling pressure was also estimated using echocardiography. We evaluated peak left ventricular filling velocity during early diastole (E wave), during atrial contraction (A wave) and deceleration time (DT). DT value < 150 ms in patients with decreased left ventricle (LV) ejection fraction was considered as a high filling pressure LV (> 25 mmHg). Furthermore, we evaluated the motion of the mitral anulus at its septal and lateral margins using pulse Doppler tissue echocardiography. As a normal diastolic velocity e‘of the lateral margin we considered ≥10 cm/s and septal ≥7 cm/s, the reduction of velocity e‘was one of the criteria of increased LV filling pressure. We calculated the E/e ‘ratio. The value of the E/e’ ratio (e’ as the average velocity of the two annular edges) ≤ 8 was indicative of the low LV filling pressure, the value > 14 reflected an increased pressure in the left atrium. If we evaluated only the rate of the lateral edge of the mitral anulus, the value > 13 was considered to be a sign of increased LV filling pressure. For the calculation using the septal margin only, the value of > 15 was considered as increased one. We considered 8–13 as a gray zone and we did not take it as a sign of increased filling pressure LV.

### Statistical analysis

Sample size calculation for Bland-Altman analyses was based on estimated mean difference 5 mmHg with SD of 10 mmHg. It was estimated that 83 pairs of independent measurements would be required for 2-tailed α of 0.5 and a 1-β of 80%. Bland-Altman analyses were performed using MedCalc Statistical Software version 18.11.3 (MedCalc Software bvba, Ostend, Belgium). SAS software version 9.4 (SAS Institute Inc., USA) was used for data management and other statistical analyses. Results are presented as the arithmetic mean ± standard deviation, median with inter-quartile range (IQR) or as a proportion (percentage); differences among groups were assessed using the paired Student’s t test, the Kruskal-Wallis test, and the χ^2^ test, respectively. We used the two-tailed test for *P* value calculations.

Agreement between two different methods of BP measurement corrected for repeated measurements in one subject, using Bland-Altman analysis [9] and concordance correlation coefficients [10], was tested. Percentage error was calculated as: (non-invasive BP – invasive BP)/invasive BP * 100.The accuracy of BP measurements was also estimated according to the British Hypertension Society (i.e., a minimum percentage of readings must be within 5, 10, and 15 mmHg. For example, to meet grade A, the absolute difference between the two methods must be less than 5 mmHg in at least 60% of measurements, less than 10 mmHg in at least 85% of measurements and less than 15 mmHg in at least 95% of measurements. To achieve grade C, the corresponding percentages are 40, 65 and 85. To achieve a grade all three percentages must be equal to or greater than the tabulated values. [11]. We also used linear regression analysis after accounting for repeated measurement in one subject to study determinants of BP differences.

## Results

### Characteristics of the study population

The characteristics of the study population are reported in Table 1. The etiology of cardiogenic shock was as follows: acute coronary syndrome (*n* = 14), cardiac arrest due to acute coronary syndrome (*n* = 40), acute decompensation of chronic heart failure (*n* = 17), severe stenosis of aortic valve (*n* = 7), endocarditis (*n* = 3), myocarditis (*n* = 2), and severe mitral regurgitation (*n* = 2).

**Table 1:** Characteristics of the study population

Variables	
n	85
Age, years	65.5 ± 12.6
Men, n (%)	66 (77.6)
Baseline characteristics	
Invasive systolic BP, mm Hg	120.2 ± 20.2
Invasive diastolic BP, mm Hg	62.8 ± 11.6
Invasive mean arterial pressure, mm Hg	82.0 ± 12.0
Hs Troponin I, ng/l	84 (51–238)
NT-proBNP, ng/l	3005 (587–8132)
Serum lactate, mmol/l	1.7 (1.2–2.3)
pH	7.33 ± 0.09
Left ventricle ejection fraction, %	31.6 ± 4.9
Glasgow coma scale, points	3 (3–14)
Mechanical ventilation, n (%)	63 (74.1)
Outcome data	
Mortality in CCU, n (%)	12 (14.1)
CPC at the time of discharge from CCU, points	1 (1–3)

Values are mean ± standard deviation, numbers (percentage) or median (interquartile range). P for difference between groups was calculated using the Student t-test, Fisher exact test, or Wilcoxon test. CPC - Cerebral Performance Categories; CCU – cardiac care unit

### Comparison of invasive and non-invasive BP measurements

**Fig. 1:**
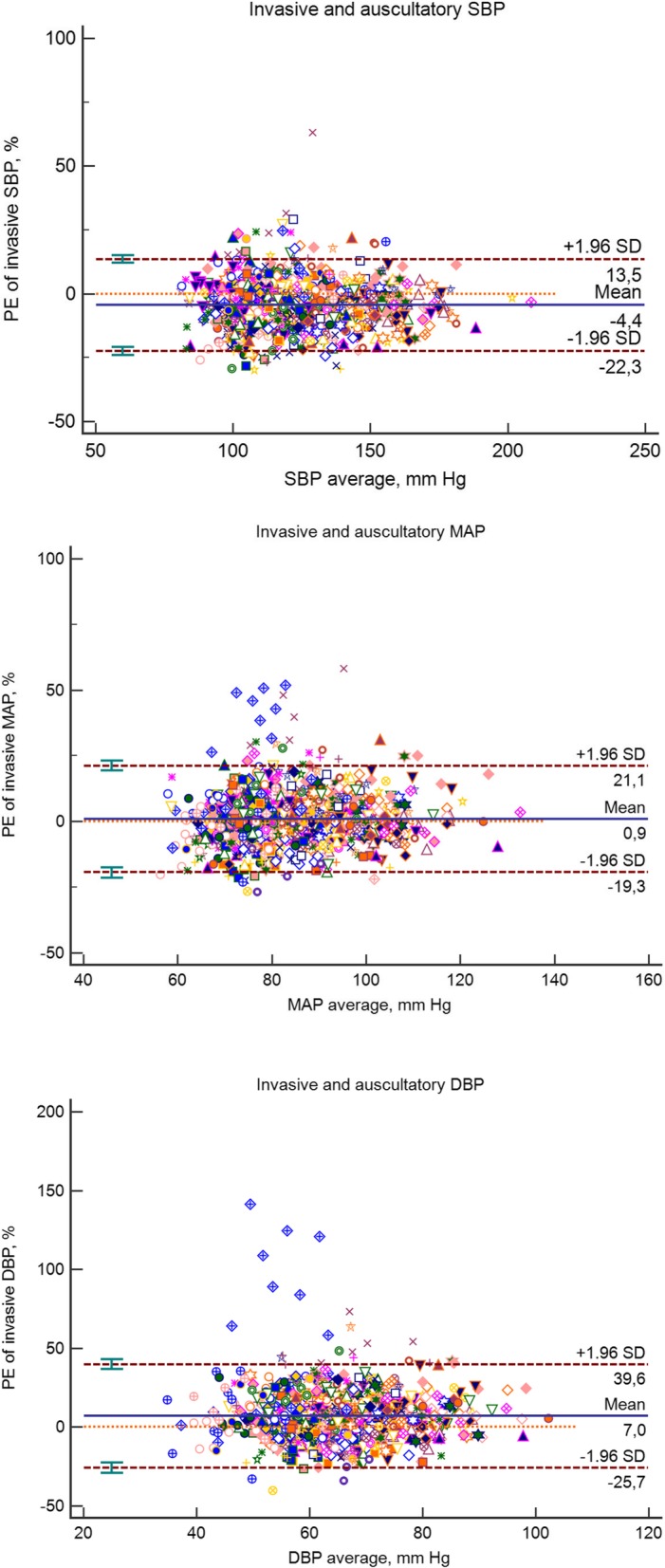
Bland – Altman plots for auscultatory vs. invasive blood pressures. Data are corrected for repeated measurements in one subject and expressed as percentage error of invasive BP. Mean differences (solid lines) and 1.96 SDs (dashed lines) are shown

**Fig. 2:**
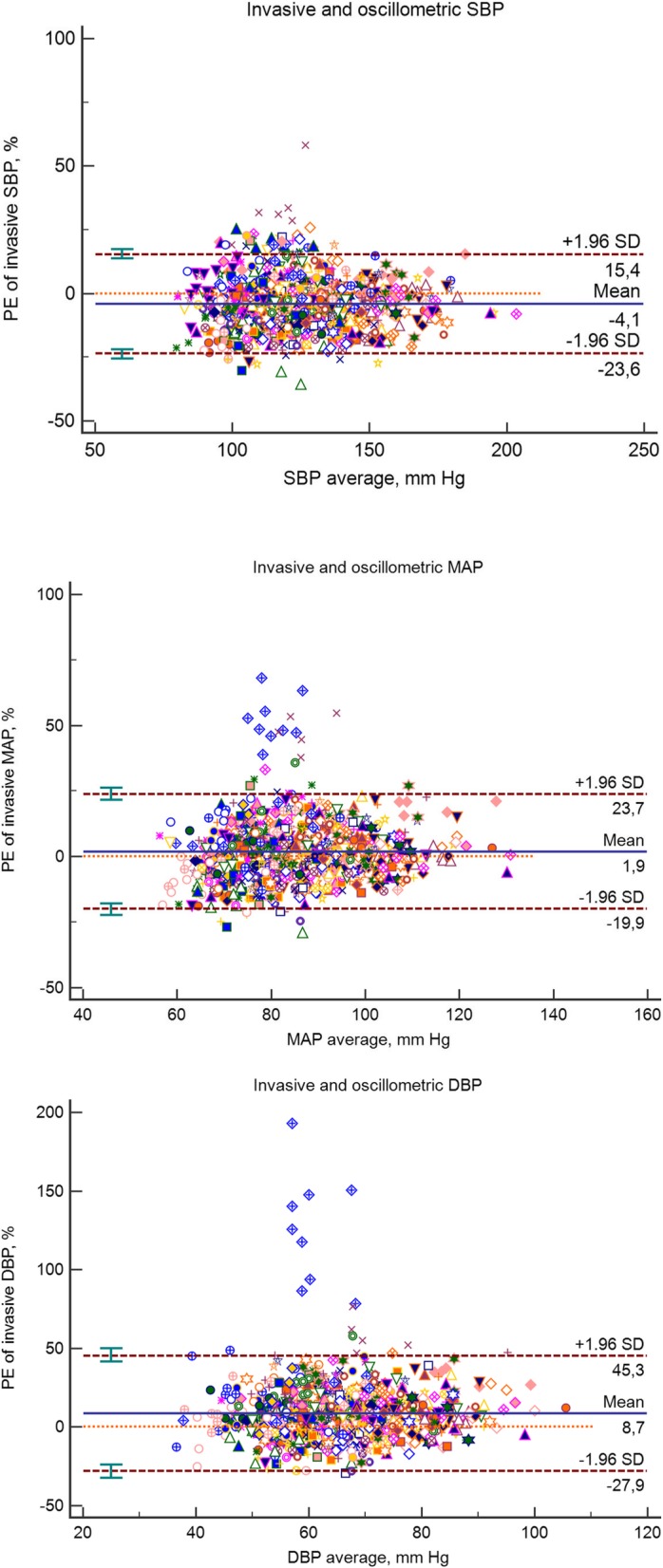
Bland – Altman plots for oscillometric vs. invasive blood pressures. Data are corrected for repeated measurements in one subject and expressed as percentage error of invasive BP. Mean differences (solid lines) and 2 1.96 SDs (dashed lines) are shown

Analyzing all 967 sets of measurements together, the auscultatory systolic blood pressure (auscSBP) and oscillometric systolic blood pressure (oscSBP) were − 6.1 and − 5.8 mmHg lower than the invasive systolic blood pressure (invSBP), respectively. On the other hand, auscultatory and oscillometric DBP (+ 3.7 and + 4.7 mmHg) and MAP (+ 0.4 and + 1.1 mmHg) were higher than the invasively measured values. Concordance correlation coefficients between invasive and auscultatory or oscillometric BP are given in Table 2. The Bland-Altman graphs accounted for repeated measurements in one subject and percentage error show large inter-individual differences between auscultatory and invasive (Fig. 1) or oscillometric and invasive (Fig. 2) BP measurements. For MAP and DBP there were clearly visible two outliers. After removing these two patients from Bland-Altman analyses, the percentage errors (95% CI) for MAP and DBP were as follows: auscultatory measurements, MAP + 0.2 (− 17.4 to 17.9) % and DBP + 5.8 (− 20.6 to 32.2) %; oscillometric measurements, MAP + 1.0 (− 17.1 to 19.1) % and DBP + 7.2 (− 19.1 to 33.4) %. Additional file 1: Figure S1 shows scatter plots of non-invasive vs. invasive measurements. Additional file 1: Table S1 shows a comparison of invasive and non-invasive BP measurements in terms of percentages of readings that varied by ≤15, ≤ 10, and ≤ 5 mmHg. According to BHS protocol [11], grade B (good) agreement was achieved only for auscultatory MAP and oscillometric MAP measurements. On the other hand, non-invasive SBP and DBP achieved a grade of D and C (very poor and poor), respectively.

**Table 2:** The relationships between invasive and auscultatory or oscillometric BP measurements (*n* = 967)

BP variables	Systolic	Diastolic	Mean
Invasive BP			
Mean ± SD, mm Hg	126.2 ± 21.3	63.2 ± 10.9	84.2 ± 12.3
Auscultatory BP			
Mean ± SD, mm Hg	120.1 ± 20.5	66.9 ± 11.6	84.6 ± 12.9
Difference (auscBP – invBP)			
Mean ± SD, mm Hg	−6.1 ± 11.4	3.7 ± 9.0	0.4 ± 8.2
Percentage error of invasive BP	−4.4 ± 9.0	7.0 ± 16.4	0.9 ± 10.2
95% limits of agreement	−22.3 to 13.5	−25.7 to 39.6	−19.3 to 21.1
CCC between invBP and auscBP	0.82 (0.80–0.84)	0.65 (0.61–0.68)	0.79 (0.76–0.81)
Oscillometric BP			
Mean ± SD	120.4 ± 20.4	67.8 ± 12.1	85.3 ± 13.3
Difference (oscBP – invBP)			
Mean ± SD	−5.8 ± 12.3	4.7 ± 9.3	1.1 ± 8.5
Percentage error of invasive BP	−4.1 ± 9.9	8.5 ± 18.2	1.8 ± 10.9
95% limits of agreement	−23.6 to 15.4	−19.9 to 23.7	−27.9 to 45.3
CCC invBP and oscBP	0.80 (0.77–0.82)	0.62 (0.59–0.66)	0.77 (0.75–0.80)

BP values are showed as means ± SD (median); BP unit is mm Hg; CCC, concordance correlation coefficients with 95% confidence intervals

In a regression analysis accounting for repeated measurements, determinants of mean bias between oscillometric and invasive measurement were invBP (β − 0.21 ± 0.02; *P* < 0.0001) and age (β − 0.07 ± 0.03; *P* = 0.018) for SBP. For MAP, the age-effect was no longer significant [BP (β − 0.17 ± 0.02; P < 0.0001) and age (β − 0.02 ± 0.02; *P* = 0.26)]. Thus, the higher invasive BP the lower oscillometric BP. Similar finding was observed for auscultatory BP.

### Impact of presence of shock on BP measurements

In a Additional file 1 Figure S2 and Table S2 we compared 289 measurements performed in patients who in that time fulfilled criteria for shock to the other measurements. BP measurements taken during shock were lower compared to those obtained when patients no longer fulfilled criteria for shock. Both subgroups achieved grade D for oscillometric systolic and grade B for mean arterial pressure according to BHS protocol [11]. Also, CCCs for systolic and mean arterial pressures tended to be lower in a presence of shock.

### Impact of therapeutic hypothermia on BP measurements

Next, we analyzed the effect of therapeutic hypothermia (in patients after cardiac arrest). While the patient’s core body temperature was lower than 36 °C 148 sets of measurements were taken. SBP and MAP were lower in hypothermic patients compared to patients with body temperatures higher than 36 °C (SBP, 118.0 ± 17.6 vs. 127.7 ± 21.6 mmHg; MAP, 81.6 ± 10.2 vs. 84.6 ± 12.5 mmHg; Additional file 1 Table S3). DBP was similar in both groups. Both subgroups achieved grade D for oscillometric systolic and grade B for mean arterial pressure according to BHS protocol [11]. In hypothermic patients, both auscultatory (CCC 0.73 [95%CI 0.64–0.77] vs. 0.79 [0.77–0.82]) and oscillometric (CCC 0.71 [0.62–0.77] vs. 0.78 [0.76–0.80]) MAP measurements agreed to a lesser extent with invasive measurements than in patients with body temperature ≥ 36 °C.

### Impact of mechanical ventilation on BP measurements

There were 737 sets of measurements taken while the patient was on mechanical ventilation. Ventilated patients had, on average, higher blood pressure values. Mechanical ventilation did not affect agreement between invasive and auscultatory BP measurements. According to BHS protocol both subgroups achieved grade D for oscillometric SBP. However, mechanically ventilated patients achieved grade B for mean arterial pressure, while spontaneously breathing patients achieved grade C [11]. The reliability of oscillometric MAP (CCC 0.80 [0.77–0.82] vs. 0.71 [0.64–0.77]) was even higher in mechanically ventilated patients compared to spontaneously breathing subjects (Additional file 1 Table S4).

### Impact of arrhythmia on BP measurements

From all measurements, 280 readings were assessed when an arrhythmia was present (atrial fibrillation in 58% of cases). Systolic and mean blood pressures where lower when arrhythmia was present. According to BHS protocol both subgroups achieved grade D for oscillometric SBP. However, patients with sinus rhythm achieved grade B for mean arterial pressure, while patients with arrhythmia present achieved grade C [11]. Moreover, presence of arrhythmia worsened the reliability of both auscultatory SBP (CCC 0.75 [0.70–0.80] vs. 0.84 [0.81–0.85]) and oscillometric SBP (CCC 0.71 [0.64–0.76] vs. 0.82 [0.79–0.84]; Additional file 1 Table S5). However, CCCs for auscBP and oscBP relative to invBP were comparable.

### Impact of severe left ventricle dysfunction on BP measurements

The maximal left ventricle ejection fraction assessed within the first twelve hour after admission was 40%. Eighteen patients, in whom 200 sets of measurement were done, had left ventricle ejection fraction (EF) lower than 30%. Patients with severely reduced EF had lower BP compared to patients with milder systolic dysfunction (Additional file 1 Table S6). Presence of severe left ventricular dysfunction did not affect grading according to BHS protocol [11], however the CCCs differed. While agreement for SBP tended to be higher in those with milder left ventricle dysfunction compared to those with severe dysfunction, for DBP and MAP we observed opposite relation. For SBP, the lower agreement in patients with severely reduced EF was given by higher mean difference between invasive and non-invasive measurement. On the other hand, in DBP and MAP the higher agreement in patients with reduce EF was driven mainly by lower variation of the difference (e.g. standard deviation). CCCs between auscBP and oscBP relative to invBP were similar.

### Impact of low BP on MAP

We further analyzed the effect of low BP on the accuracy of non-invasive BP measurements. In 80 BP readings, when invSBP was below 100 mmHg, the accuracy of non-invasive MAP was very low (e.g. oscillometric, CCC 0.36 [95% CI 0.00–0.64] compared to readings with invSBP> 100 mmHg, CCC 0.79 [95%CI 0.75–0.82]); Addijtional file 1 FigureS1). As above mentioned clinical situations which tended to achieve lower reliability assessed as CCC had in general lower BP, it is quite possible that underlying mechanism for this observation is indeed the level of blood pressure (Additional file 1 Table S7).

## Discussion

In our study we showed that in patients admitted for cardiac shock, non-invasive BP measurements are often inaccurate compared to invasive measurements, mainly with regards to systolic BP. Although concordance correlation coefficients between non-invasive and invasive BP were relatively high and the mean bias were relatively low, there was large, clinically potentially important, variability among individuals (2 SDs for systolic, diastolic, and mean blood pressure were 23, 18, and 17 mmHg, respectively). Only MAP reached good agreement according British Hypertension Society protocol [11]. Moreover, we observed that clinical conditions generally associated with hypotension affected the agreement between non-invasive and invasive BP (e.g., severe hypotension and therapeutic hypothermia). Auscultatory and oscillometric measurements had similar reliability, even in patients with arrhythmia.

Lehman et al. recently used a large ICU database to compare invasive arterial and oscillometric BP and demonstrated clinically significant discrepancies between the methods [6]. In concordance with our and other results [7], non-invasive BP tended to be higher than invasive BP at low blood pressures and lower than invasive BP at high blood pressures. One explanation for this observation might be pressure augmentation. Indeed, peripheral vasoconstriction caused either due to hypotension itself or induced by pharmacological vasoconstrictors, can lead to an increase in wave reflection and thus augmentation of central SBP. Moreover, differences between systolic non-invasive (brachial, thus more central) and invasive (radial, thus more peripheral) BP were negatively associated (independently of underlying BP) with age (which supports the effect of arterial stiffness) and changes in reflection sites during ageing.

In our settings, mean bias for systolic and diastolic pressures were in agreement with results of recently published meta-analysis comparing non-invasively and invasively measured blood pressure in more than 3000 subjects [12]. We observed that oscillometric SBP were 5.8 mmHg lower and DBP 4.7 mmHg higher than invasively obtained values. Picone et al. reported mean bias accounting to − 5.7 mmHg and 5.5 mmHg. Also percentages of readings with absolute difference between the two methods less than 5, 10 and 15 mmHg were comparable in our study and Picone’s meta-analysis (see. Additonal file 1 Table S1).

Mean arterial pressure reflects perfusion pressure and is the main hemodynamic parameter monitored by the neurohormonal system to control BP. During treatment of shock, MAP is the key pressure. In our study we showed that in hypotensive patients (invSBP < 100 mmHg), the mean pressure values obtained by both auscBP and oscBP were inaccurate (Additional file 1 Figure S1). This finding is clinically significant. In the initial management of shock, we rely on non-invasively measured BP (before other methods are started). Therefore, in patients with relatively sufficient non-invasive BP, but with clinical signs of hypotension, we must be aware that real MAP values could be very different. It should be pointed out that in severe hypotension, the oscillometric tonometers sometimes fail to measure BP at all.

Moreover, we observed that in patients with therapeutic hypothermia, agreement between invasive and non-invasive MAP was lower than in subjects with a body temperature higher than 36 °C. The probable explanation for this finding is accentuated vasoconstriction during hypothermia (and also during severe hypotension as mentioned above), which leads to a rise in BP, per se, but also affects radial-brachial pressure relationships [13].

We also observed that mechanical ventilation did not worsen agreement between invasive and non-invasive BP measurement. On the contrary, in mechanically ventilated patients, agreement between invasive and oscillometric (but not auscultatory) DBP and MAP was higher than in spontaneously breathing patients. Mechanical ventilation leads to changes in intrathoracic pressure and thus directly affects monitored BP [3]. Since current monitoring systems determine BP readings at predetermined intervals, and not at the end of expiration, respiratory artifacts can lead to erroneous digital output [3]. However, to minimize this effect of this phenomenon in our study, invBP values were taken at the end of expiration.

Moreover, we did not observe lower agreement between oscillometric BP and invBP compared to auscultatory BP. Although influence of an irregular heart rhythm on the reliability of oscillometric BP measurements is well known and generally accepted [14], several authors suggest that repeated measurements in one session (as we did in our study) might reduce this source of error [2].

### Limitations and strengths

First, this was a single-center study on a limited number of patients. However, we applied strict quality control of BP measurements and only nine of the most experienced nurses (shift supervisors) performed all BP measurements. Moreover, we used a dual BP tonometer (DM-3000) for both auscultatory and oscillometric measurements. Therefore, we eliminated measurement errors that might have resulted from different types of arm cuffs or cuff positioning. Second, our results, which are based on patients admitted for cardiac shock, might not be extrapolatable to the general population or other critically ill patients. Third, we used calculated non-invasive MAPs (from systolic and diastolic BP values) for our analyses. However, we tried to mirror frequent clinical practice as majority of oscillometric tonometers do not give MAP value. It was shown that calculated MAP using broadly used 1/3 rule underestimates real MAP [15, 16]. In our patients we observed much lower mean bias between non-invasive and invasive MAP but with very large variability. It is likely that the hemodynamic factors differ in patient with shock and estimation of MAP is even more imprecise. Fourth, invasive and non-invasive BPs were measured at different anatomical sites (radial vs. brachial artery), however, this reflects common clinical practice. Nonetheless, some of the discrepancies in BP values might be explained by this factor. And fifth, in the present analysis we did not study the effect of vasoactive drugs on BP measurements. This will be part of the subsequent analysis.

## Conclusions

Mean arterial pressure (the key pressure in critically-ill patients) measured non-invasively appears to be in good agreement with invasively measured MAP in patients with cardiogenic shock. From a practical point of view, it is important to acknowledge that several clinical conditions associated with hypotension affect the accuracy of non-invasive MAP or SBP measurements, e.g., presence of shock and therapeutic hypothermia. In these situations, use of invasive measurements should be recommended.

## Additional files

Additional file 1: **Figure S1.** Scatter plots for auscultatory vs. invasive measurements (left panels) and oscillometric vs. invasive (right panels) blood pressure measurements. **Figure S2.** Effect of severe hypotension on accuracy of non-invasive MAP measurements. Upper panels show scatter plots between invasive and non-invasive measurements in those cases where invSBP < 100 mmHg (*n* = 80), lower panels for those with invSBP ≥ than 100 mmHg (*n* = 887). **Table S1.** BHS grade of agreement between noninvasive and invasive methods: cumulative percentage of absolute difference (mmHg) between invasive BP and other studied methods (auscBP and oscBP). **Table S2.** Effect of current cardiogenic shock on accuracy of non-invasive BP measurement. **Table S3.** Effect of therapeutic hypothermia on accuracy of non-invasive BP measurement. **Table S4.** Effect of mechanical ventilation on accuracy of non-invasive BP measurement. **Table S5.** Effect of arrhythmia on accuracy of non-invasive BP measurement. **Table S6.** Effect of severe left ventricular dysfunction on accuracy of non-invasive BP measurement. **Table S7.** Frequency of potential factors affecting accuracy of non-invasive BP measurement in respect to hypotension (invSBP<100mmHg). (DOCX 464 kb)

## Data Availability

The datasets generated and/or analyzed during the current study are not publicly available due to the fact, that this was not part of the informed consent and was not approved by the local Ethical committee but are available from the corresponding author on reasonable request.

## Abbreviations

auscDBP: Auscultatory diastolic blood pressure
auscMAP: Auscultatory mean arterial pressure
auscSBP: Auscultatory systolic blood pressure
BP: Blood pressure
CCC: Concordance correlation coefficients
CCU: Coronary care unit
DBP: Diastolic blood pressure
DT: Deceleration time
EF: Ejection fraction
ICU: Intensive care unit
invBP: Invasively measured BP
invDBP: Invasive diastolic blood pressure
invMAP: Invasive mean arterial pressure
invSBP: Invasive systolic blood pressure
LV: Left ventricle
oscDBP: Oscillometric diastolic blood pressure
oscMAP: Oscillometric mean arterial pressure
oscSBP: Oscillometric systolic blood pressure
RASS: Richmond agitation and sedation scale
SBP: Systolic blood pressure
