# Supplementary material for: Factors influencing the accuracy of non-invasive blood pressure measurements in patients admitted for cardiogenic shock
Source: BMC Cardiovasc Disord. 2019 Jun 18;19:150. doi: 10.1186/s12872-019-1129-9 (PMC6582540; doi:10.1186/s12872-019-1129-9)
Supplement: Supplementary file 1 — Figure S1. Scatter plots for auscultatory vs. invasive measurements (left panels) and oscillometric vs. invasive (right panels) blood pressure measurements. Figure S2. Effect of severe hypotension on accuracy of non-invasive MAP measurements. Upper panels show scatter plots between invasive and non-invasive measurements in those cases where invSBP < 100 mmHg (n = 80), lower panels for those with invSBP ≥ than 100 mmHg (n = 887). Table S1. BHS grade of agreement between noninvasive and invasive methods: cumulative percentage of absolute difference (mmHg) between invasive BP and other studied methods (auscBP and oscBP). Table S2. Effect of current cardiogenic shock on accuracy of non-invasive BP measurement. Table S3. Effect of therapeutic hypothermia on accuracy of non-invasive BP measurement. Table S4. Effect of mechanical ventilation on accuracy of non-invasive BP measurement. Table S5. Effect of arrhythmia on accuracy of non-invasive BP measurement. Table S6. Effect of severe left ventricular dysfunction on accuracy of non-invasive BP measurement. Table S7. Frequency of potential factors affecting accuracy of non-invasive BP measurement in respect to hypotension (invSBP<100mmHg). (DOCX 464 kb) [file 12872_2019_1129_MOESM1_ESM.docx]

**Supplemental Materials**

**Factors influencing the accuracy of noninvasive blood pressure measurements in patients admitted for cardiogenic shock**

**Supplemental Table 1** BHS grade of agreement between noninvasive and invasive methods: cumulative percentage of absolute difference (mmHg) between invasive BP and other studied methods (auscBP and oscBP).

|  | ≤ 5 mm Hg | ≤ 10 mm Hg | ≤ 15 mm Hg | Grade |
| --- | --- | --- | --- | --- |
| Invasive vs. auscultatory | | | | |
| Systolic | 35 | 61 | 78 | D very poor |
| Diastolic | 45 | 74 | 90 | C poor |
| Mean | 52 | 82 | 94 | B good |
| Invasive vs. oscillometric | | | | |
| Systolic | 33 | 56 | 74 | D very poor |
| Diastolic | 46 | 74 | 88 | C poor |
| Mean | 53 | 79 | 94 | B good |

Grades are derived from percentages of readings within 5, 10, and 15mmHg: to achieve a grade, all three percentages must be equal to or greater than the tabulated values. For example, to achieve the “A” grade, sixty percent of the measured BP values with invBP and auscBP must be within 5mmHg, 85% within 10mmHg, and 95% within 15mmHg.

**Supplemental Table 2** Effect of current cardiogenic shock on accuracy of non-invasive BP measurement

| Blood pressure |  | Systolic | | Diastolic | | Mean | |
| --- | --- | --- | --- | --- | --- | --- | --- |
| Cardiogenic shock |  | Yes (n=289) | No (n=678) | Yes (n=289) | No (n=678) | Yes (n=289) | No (n=678) |
| Invasive | BP, mm Hg | 118.7±17.5 | 129.4±22.0 | 61.0±9.1 | 64.1±11.4 | 80.3±9.6 | 85.8±12.9 |
|  | P _BP values_ | <0.0001 | | <0.0001 | | <0.0001 | |
| Auscultatory | BP, mm Hg | 113.7±17.9 | 122.9±21.0 | 64.4±10.3 | 67.9±12.0 | 80.8±11.2 | 86.3±13.3 |
|  | Mean diff. ± SD | -5.0±11.3 | -6.5±11.4 | 3.4±8.5 | 3.9±9.2 | 0.5±7.9 | 0.4±8.3 |
|  | PE | -3.9±9.8 | -4.6±8.7 | 6.3±14.2 | 7.3±17.2 | 1.0±10.0 | 0.9±10.2 |
|  | (95% CI) | -23.0 to 15.2 | -21.7 to 12.5 | -21.5 to 34.1 | -26.5 to 41.1 | -18.7 to 20.6 | -19.2 to 21.0 |
|  | CCC | 0.76 | 0.82 | 0.58 | 0.66 | 0.71 | 0.80 |
|  | (95%CI) | 0.71-0.81 | 0.80-0.84 | 0.50-0.65 | 0.61-0.70 | 0.65-0.76 | 0.77-0.82 |
| Oscillometric | BP, mm Hg | 114.4±18.2 | 122.9±20.8 | 65.3±10.5 | 68.9±12.6 | 81.7±11.5 | 86.9±13.7 |
|  | Mean diff. ± SD | -4.1±12.7 | -6.5±12.1 | 4.3±8.1 | 4.8±9.7 | 1.5±8.0 | 1.0±8.7 |
|  | PE | -3.1±11.0 | -4.6±9.3 | 7.7±14.4 | 8.9±19.6 | 2.1±10.4 | 1.7±11.2 |
|  | (95% CI) | -24.6 to 18.5 | -22.8 to 13.7 | -20.4 to 35.9 | -29.5 to 47.3 | -18.3 to 22.5 | -20.2 to 23.6 |
|  | CCC | 0.73 | 0.80 | 0.60 | 0.62 | 0.71 | 0.78 |
|  | (95%CI) | 0.67-0.78 | 0.78-0.83 | 0.53-0.66 | 0.58-0.66 | 0.65-0.76 | 0.75-0.81 |

PE, percentage error of invasive BP; CCC, concordance correlation coefficient; 95% CI, 95% confidence limits. P for difference between BP measurements in patients fulfilling criteria for cardiogenic shock and others.

**Supplemental Table 3** Effect of therapeutic hypothermia on accuracy of non-invasive BP measurement

| Blood pressure |  | Systolic | | Diastolic | | Mean | |
| --- | --- | --- | --- | --- | --- | --- | --- |
| Ther. hypothermia |  | Yes (n=148) | No (n=819) | Yes (n=148) | No (n=819) | Yes (n=148) | No (n=819) |
| Invasive | BP, mm Hg | 118.0±17.6 | 127.7±21.6 | 63.4±9.8 | 63.1±11.1 | 81.6±10.2 | 84.6±12.5 |
|  | P _BP values_ | <0.0001 | | 0.72 | | 0.0018 | |
| Auscultatory | BP, mm Hg | 110.9±17.3 | 121.8±20.6 | 65.0±10.4 | 67.2±11.8 | 80.3±10.8 | 85.4±13.1 |
|  | Mean diff. ± SD | -7.1±9.3 | -5.8±11.7 | +1.5±8.9 | +4.1±8.9 | -1.4±7.6 | +0.8±8.2 |
|  | PE | -5.9±7.5 | -4.1±9.3 | +3.1±14.1 | +7.7±16.7 | -1.4±9.2 | +1.4±10.3 |
|  | (95% CI) | -20.6 to 8.9 | -22.3 to 14.0 | -24.6 to 30.7 | -24.9 to 40.4 | -19.5 to 16.7 | -18.8 to 21.5 |
|  | CCC | 0.79 | 0.81 | 0.61 | 0.65 | 0.73 | 0.79 |
|  | (95%CI) | 0.73-0.84 | 0.79-0.84 | 0.49-0.70 | 0.61-0.69 | 0.64-0.77 | 0.77-0.82 |
| Oscillometric | BP, mm Hg | 111.4±17.7 | 122.0±20.4 | 65.5±10.0 | 68.2±12.4 | 80.8±10.8 | 86.2±13.5 |
|  | Mean diff. ± SD | -6.6±10.4 | -5.6±12.6 | +2.1±8.5 | +5.1±9.3 | -0.8±7.9 | +1.5±8.6 |
|  | PE | -5.4±8.6 | -3.9±10.0 | +4.0±14.0 | +9.3±18.7 | -0.6±9.8 | +2.2±11.1 |
|  | (95% CI) | -22.2 to 11.4 | -23.6 to 15.8 | -23.4 to 31.5 | -27.4 to 46.1 | -19.9 to 18.6 | -19.5 to 24.0 |
|  | CCC | 0.77 | 0.79 | 0.62 | 0.62 | 0.71 | 0.78 |
|  | (95%CI) | 0.70-0.83 | 0.77-0.81 | 0.51-0.71 | 0.59-0.66 | 0.62-0.77 | (0.76-0.80 |

PE, percentage error of invasive BP; CCC, concordance correlation coefficient; 95% CI, 95% confidence limits; P for difference between BP measurements in patients with therapeutic hypothermia and patients with normal body temperature.

**Supplemental Table 4** Effect of mechanical ventilation on accuracy of non-invasive BP measurement

| Blood pressure |  | Systolic | | | Diastolic | | Mean | |
| --- | --- | --- | --- | --- | --- | --- | --- | --- |
| Mechanical ventilation |  | Yes (n=737) | | No (n=230) | Yes (n=737) | No (n=230) | Yes (n=737) | No (n=230) |
| Invasive | BP, mm Hg | 127.1±21.2 | | 123.4±21.5 | 64.4±10.0 | 59.1±12.6 | 85.3±11.8 | 80.5±12.8 |
|  | P _BP values_ | 0.024 | | | <0.0001 | | <0.0001 | |
| Auscultatory | BP, mm Hg | 120.4±20.3 | 119.2±21.1 | | 67.7±11.0 | 64.1±12.9 | 85.3±12.6 | 82.5±13.6 |
|  | Mean diff. ± SD | -6.6±11.3 | -4.2±11.3 | | 3.3±8.5 | 5.0±10.3 | 0±7.8 | +1.9±9.2 |
|  | PE | -4.8±8.9 | -3.0±9.2 | | 5.9±13.7 | 10.6±22.6 | 0.3±9.4 | 3.0±12.2 |
|  | (95% CI) | -22.4 to 12.7 | -21.0 to 15.0 | | -20.9 to 32.7 | -33.8 to 55.0 | -18.1 to 18.7 | -20.9 to 26.9 |
|  | CCC | 0.81 | 0.84 | | 0.64 | 0.62 | 0.80 | 0.75 |
|  | (95%CI) | 0.78-0.83 | 0.81-0.88 | | 0.60-0.68 | 0.54-0.69 | 0.77-0.82 | 0.69-0.80 |
| Oscillometric | BP, mm Hg | 120.6±19.9 | 119.7±21.8 | | 68.3±11.5 | 66.2±13.6 | 85.7±12.9 | 84.1±14.4 |
|  | Mean diff. ± SD | -6.5±12.4 | -3.7±11.9 | | 3.9±8.3 | 7.1±11.5 | 0.4±7.9 | 3.5±10.0 |
|  | PE | -4.6±9.9 | -2.6±9.7 | | 6.7±13.5 | 14.5±27.6 | 0.8±9.7 | 5.0±13.8 |
|  | (95% CI) | -23.9 to 14.8 | -21.6 to 16.3 | | -19.9 to 33.2 | -39.5 to 68.6 | -18.2 to 19.8 | -22.0 to 32.0 |
|  | CCC | 0.78 | 0.84 | | 0.66 | 0.54 | 0.80 | 0.71 |
|  | (95%CI) | 0.75-0.81 | 0.80-0.88 | | 0.62-0.77 | 0.45-0.61 | 0.77-0.82 | 0.64-0.77 |

PE, percentage error of invasive BP; CCC, concordance correlation coefficient; 95% CI, 95% confidence limits; P for difference between BP measurements between BP measurements in mechanically ventilated and spontaneously breathing patients.

**Supplemental Table 5** Effect of arrhythmia on accuracy of non-invasive BP measurement

| Blood pressure |  | Systolic | | | Diastolic | | Mean | |
| --- | --- | --- | --- | --- | --- | --- | --- | --- |
| Arrhythmia |  | Yes (n=280) | | No (n=687) | Yes (n=280) | No (n=687) | Yes (n=280) | No (n=687) |
| Invasive | BP, mm Hg | 121.7±18.4 | | 128.0±22.2 | 63.1±9.7 | 63.2±11.4 | 82.6±10.8 | 84.8±12.7 |
|  | P _BP values_ | <0.0001 | | | 0.95 | | 0.0080 | |
| Auscultatory | BP, mm Hg | 116.4±18.1 | 121.7±21.3 | | 65.8±10.7 | 67.3±11.9 | 82.7±11.7 | 85.4±13.3 |
|  | Mean diff. ± SD | -5.3±12.0 | -6.4±11.1 | | 2.7±8.1 | 4.1±9.3 | 0.1±7.8 | 0.6±8.3 |
|  | PE | -3.9±9.8 | -4.6±8.7 | | 5.0±13.3 | 7.8±17.4 | 0.4±9.7 | 1.1±10.4 |
|  | (95% CI) | -23.1 to 15.3 | -21.7 to 12.5 | | -21.1 to 31.1 | -26.3 to 41.9 | -18.6 to 19.4 | -19.2 to 21.5 |
|  | CCC | 0.75 | 0.84 | | 0.66 | 0.64 | 0.76 | 0.80 |
|  | (95%CI) | (0.70-0.80) | (0.81-0.85) | | (0.60-0.72) | (0.60-0.68) | (0.70-0.80) | (0.77-0.82) |
| Oscillometric | BP, mm Hg | 115.8±11.6 | 122.2±21.2 | | 66.5±11.5 | 68.3±12.3 | 83.0±12.0 | 86.3±13.6 |
|  | Mean diff. ± SD | -5.8±12.9 | -5.8±12.0 | | 3.4±8.5 | 5.2±9.5 | 0.3±8.2 | 1.5±8.7 |
|  | PE | -4.2±10.5 | -4.1±9.6 | | 6.0±14.0 | 9.6±19.5 | 0.7±10.1 | 2.2±11.2 |
|  | (95% CI) | -24.9 to 16.4 | -22.8 to 14.7 | | -21.5 to 33.5 | -28.7 to 47.9 | -19.1 to20.5 | -19.8 to 24.3 |
|  | CCC | 0.71 | 0.82 | | 0.65 | 0.62 | 0.74 | 0.78 |
|  | (95%CI) | (0.64-0.76) | (0.79-0.84) | | (0.58-0.71) | (0.57-0.66) | (0.69-0.79) | (0.75-0.81) |

PE, percentage error of invasive BP; CCC, concordance correlation coefficient; 95% CI, 95% confidence limits; P for difference between BP measurements between BP measurements in patients with arrhythmia and with regular sinus rhythm.

**Supplemental Table 6** Effect of severe left ventricular dysfunction on accuracy of non-invasive BP measurement

| Blood pressure |  | Systolic | | | Diastolic | | Mean | |
| --- | --- | --- | --- | --- | --- | --- | --- | --- |
| EF < 30% | | Yes (n=200) | | No (n=767) | Yes (n=200) | No (n=767) | Yes (n=200) | No (n=767) |
| Invasive | BP, mm Hg | 122.6±17.7 | | 127.1±22.1 | 60.5±11.0 | 63.8±10.8 | 81.2±11.6 | 84.9±12.3 |
|  | P _BP values_ | 0.0033 | | | <0.0001 | | <0.0001 | |
| Auscultatory | BP, mm Hg | 115.5±17.4 | 121.4±21.1 | | 64.5±11.0 | 67.5±11.7 | 81.5±12.1 | 85.4±13.0 |
|  | Mean diff. ± SD | -7.2±10.3 | -5.7±11.6 | | 4.0±7.4 | 3.7±9.4 | 0.3±6.8 | 0.5±8.5 |
|  | PE | -5.6±8.5 | -4.1±9.2 | | 7.4±12.7 | 6.9±17.2 | 0.6±8.6 | 1.0±10.6 |
|  | (95% CI) | -22.2 to 11.0 | -22.1 to 13.9 | | -17.4 to 32.3 | -26.9 to 40.6 | -16.2 to 17.4 | -19.7 to 21.7 |
|  | CCC | 0.76 | 0.83 | | 0.73 | 0.62 | 0.83 | 0.76 |
|  | (95%CI) | (0.71-0.81) | (0.80-0.85) | | (0.66-0.78) | (0.58-0.66) | (0.79-0.87) | (0.72-0.78) |
| Oscillometric | BP, mm Hg | 115.7±17.1 | 121.6±21.0 | | 65.5±11.6 | 68.4±12.2 | 82.3±12.3 | 86.1±13.4 |
|  | Mean diff. ± SD | -6.9±11.3 | -5.5±12.5 | | 5.0±7.2 | 4.6±9.8 | 1.0±6.8 | 1.2±8.9 |
|  | PE | -5.3±9.3 | -3.8±10.0 | | 8.9±12.5 | 8.4±19.4 | 1.5±8.8 | 1.9±11.4 |
|  | (95% CI) | -23.5 to 13.0 | -23.4 to 15.7 | | -15.6 to 33.5 | -29.6 to 46.5 | -15.7 to 18.7 | -20.6 to 24.3 |
|  | CCC | 0.73 | 0.80 | | 0.73 | 0.59 | 0.83 | 0.75 |
|  | (95%CI) | (0.66-0.79) | (0.78-0.83) | | (0.66-0.78) | (0.55-0.63) | (0.79-0.87) | (0.71-0.78) |

PE, percentage error of invasive BP; CCC, concordance correlation coefficient; 95% CI, 95% confidence limits; P for difference between BP measurements in patients with severe left ventricular systolic dysfunction compared to patients with milder forms of left ventricle dysfunction (ejection fraction 30-40%).**Supplemental Table 7** Frequency of potential factors affecting accuracy of non-invasive BP measurement in respect to hypotension (invSBP<100mmHg)

|  | InvSBP<100 mm Hg n=80 | InvSBP>100 mm Hg n=887 | P |
| --- | --- | --- | --- |
| Presence of shock, n (%) | 36 (45.0) | 253 (28.5) | 0.0031 |
| Therapeutic hypothermia, n (%) | 18 (22.5) | 130 (14.7) | 0.073 |
| UPV, n (%) | 49 (61.2) | 688 (77.6) | 0.0023 |
| Arrhythmia, n (%) | 27 (33.7) | 253 (28.5) | 0.37 |
| Severe left ventricular dysfunction, n (%) | 11 (13.7) | 189 (21.3) | 0.11 |

P for difference between the two groups was calculated using Fisher’s Exact test.

##
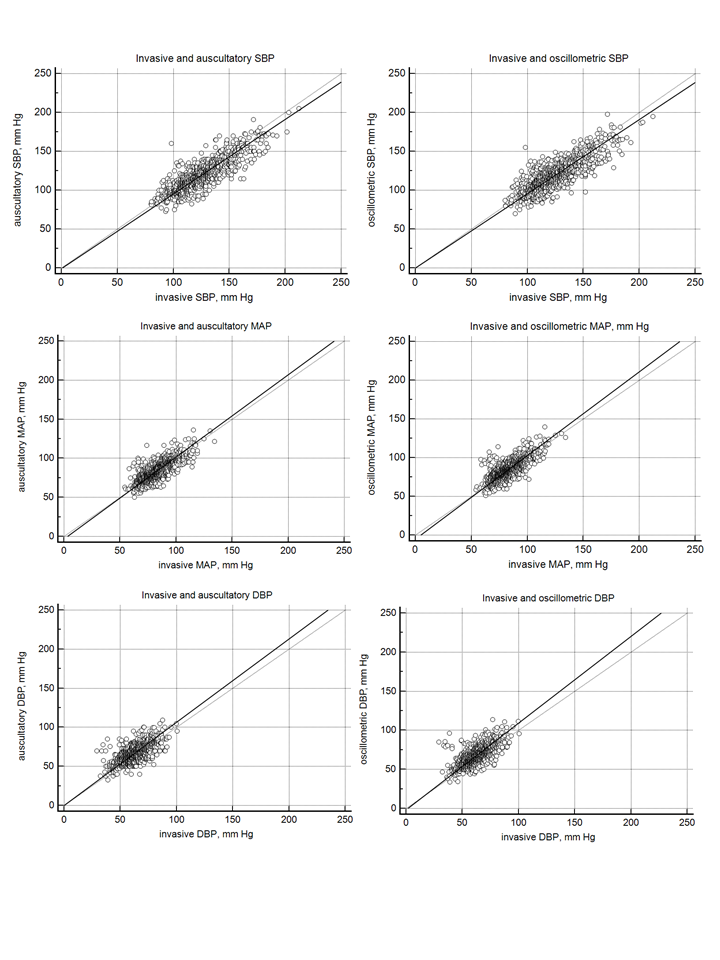


## Supplemental Figure 1 Supplemental Figure 1 Scatter plots for auscultatory vs. invasive measurements (left panels) and oscillometric vs. invasive (right panels) blood pressure measurements.


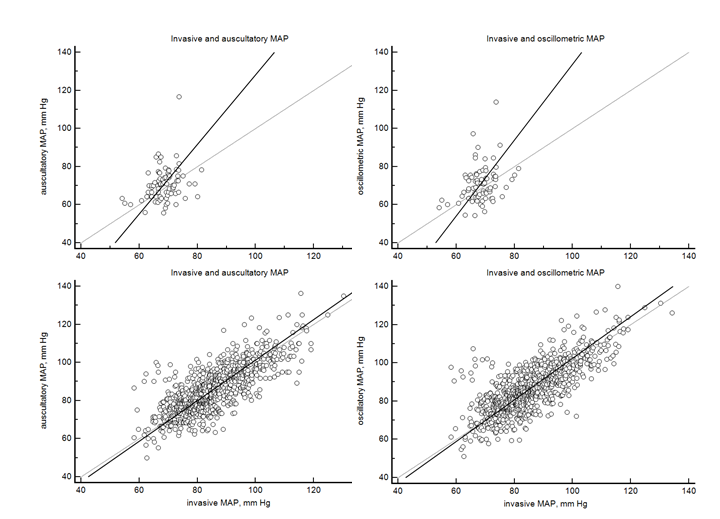


## Supplemental Figure 2 Effect of severe hypotension on accuracy of non-invasive MAP measurements. Upper panels show scatter plots between invasive and non-invasive measurements in those cases where invSBP < 100 mm Hg (n=80), lower panels for those with invSBP ≥ than 100 mm Hg (n=887).
